# Supplementary material for: Unveiling the Bioactive Potential of the Invasive Jellyfish Phyllorhiza punctata Through Integrative Transcriptomic and Proteomic Analyses
Source: Biomolecules. 2025 Aug 4;15(8):1121. doi: 10.3390/biom15081121 (PMC12383608; doi:10.3390/biom15081121)
Supplement: Supplementary file 1 [file biomolecules-15-01121-s001.zip › Suplementary Material/FileS3/PEPFOLD4/XP_065055734_6717/clusters.html]

 PEP-FOLD clusters


Download archive of all models  here   

|  |  |  |
| --- | --- | --- |
| # | file | sOPEP |
| # cluster 1 - Size: 1 |  |  |
|  | XP\_065055734\_6717-model1 | -10.4592 |
| # cluster 2 - Size: 3 |  |  |
|  | XP\_065055734\_6717-model2 | -10.4273 |
|  | XP\_065055734\_6717-model2.1 | -9.75953 |
|  | XP\_065055734\_6717-model2.2 | -9.69802 |
| # cluster 3 - Size: 1 |  |  |
|  | XP\_065055734\_6717-model3 | -10.1743 |
| # cluster 4 - Size: 21 |  |  |
|  | XP\_065055734\_6717-model4 | -9.96353 |
|  | XP\_065055734\_6717-model4.1 | -9.96353 |
|  | XP\_065055734\_6717-model4.2 | -9.8026 |
|  | XP\_065055734\_6717-model4.3 | -9.8026 |
|  | XP\_065055734\_6717-model4.4 | -9.8026 |
|  | XP\_065055734\_6717-model4.5 | -9.57475 |
|  | XP\_065055734\_6717-model4.6 | -9.40962 |
|  | XP\_065055734\_6717-model4.7 | -9.39355 |
|  | XP\_065055734\_6717-model4.8 | -9.37104 |
|  | XP\_065055734\_6717-model4.9 | -9.13927 |
|  | XP\_065055734\_6717-model4.10 | -9.11262 |
|  | XP\_065055734\_6717-model4.11 | -9.08847 |
|  | XP\_065055734\_6717-model4.12 | -9.06114 |
|  | XP\_065055734\_6717-model4.13 | -8.64567 |
|  | XP\_065055734\_6717-model4.14 | -8.16465 |
|  | XP\_065055734\_6717-model4.15 | -8.09138 |
|  | XP\_065055734\_6717-model4.16 | -7.90119 |
|  | XP\_065055734\_6717-model4.17 | -7.47022 |
|  | XP\_065055734\_6717-model4.18 | -7.12558 |
|  | XP\_065055734\_6717-model4.19 | -7.01373 |
|  | XP\_065055734\_6717-model4.20 | -6.77332 |
| # cluster 5 - Size: 35 |  |  |
|  | XP\_065055734\_6717-model5 | -9.83307 |
|  | XP\_065055734\_6717-model5.1 | -9.83307 |
|  | XP\_065055734\_6717-model5.2 | -9.76318 |
|  | XP\_065055734\_6717-model5.3 | -9.75064 |
|  | XP\_065055734\_6717-model5.4 | -9.62746 |
|  | XP\_065055734\_6717-model5.5 | -9.6186 |
|  | XP\_065055734\_6717-model5.6 | -9.29627 |
|  | XP\_065055734\_6717-model5.7 | -9.27995 |
|  | XP\_065055734\_6717-model5.8 | -9.25707 |
|  | XP\_065055734\_6717-model5.9 | -9.17597 |
|  | XP\_065055734\_6717-model5.10 | -9.16263 |
|  | XP\_065055734\_6717-model5.11 | -9.14571 |
|  | XP\_065055734\_6717-model5.12 | -8.97061 |
|  | XP\_065055734\_6717-model5.13 | -8.94634 |
|  | XP\_065055734\_6717-model5.14 | -8.94634 |
|  | XP\_065055734\_6717-model5.15 | -8.8361 |
|  | XP\_065055734\_6717-model5.16 | -8.67004 |
|  | XP\_065055734\_6717-model5.17 | -8.65007 |
|  | XP\_065055734\_6717-model5.18 | -8.52809 |
|  | XP\_065055734\_6717-model5.19 | -8.50659 |
|  | XP\_065055734\_6717-model5.20 | -8.50054 |
|  | XP\_065055734\_6717-model5.21 | -8.46129 |
|  | XP\_065055734\_6717-model5.22 | -8.40568 |
|  | XP\_065055734\_6717-model5.23 | -8.33891 |
|  | XP\_065055734\_6717-model5.24 | -8.31336 |
|  | XP\_065055734\_6717-model5.25 | -8.29729 |
|  | XP\_065055734\_6717-model5.26 | -8.27418 |
|  | XP\_065055734\_6717-model5.27 | -8.22474 |
|  | XP\_065055734\_6717-model5.28 | -8.21848 |
|  | XP\_065055734\_6717-model5.29 | -8.21843 |
|  | XP\_065055734\_6717-model5.30 | -8.21782 |
|  | XP\_065055734\_6717-model5.31 | -7.99023 |
|  | XP\_065055734\_6717-model5.32 | -7.79041 |
|  | XP\_065055734\_6717-model5.33 | -7.74839 |
|  | XP\_065055734\_6717-model5.34 | -6.68924 |
| # cluster 6 - Size: 2 |  |  |
|  | XP\_065055734\_6717-model6 | -9.61021 |
|  | XP\_065055734\_6717-model6.1 | -9.33862 |
| # cluster 7 - Size: 12 |  |  |
|  | XP\_065055734\_6717-model7 | -9.24209 |
|  | XP\_065055734\_6717-model7.1 | -9.01509 |
|  | XP\_065055734\_6717-model7.2 | -8.60368 |
|  | XP\_065055734\_6717-model7.3 | -8.49527 |
|  | XP\_065055734\_6717-model7.4 | -8.49527 |
|  | XP\_065055734\_6717-model7.5 | -8.26513 |
|  | XP\_065055734\_6717-model7.6 | -7.8381 |
|  | XP\_065055734\_6717-model7.7 | -7.80557 |
|  | XP\_065055734\_6717-model7.8 | -6.96288 |
|  | XP\_065055734\_6717-model7.9 | -6.48729 |
|  | XP\_065055734\_6717-model7.10 | -5.8464 |
|  | XP\_065055734\_6717-model7.11 | -5.83851 |
| # cluster 8 - Size: 6 |  |  |
|  | XP\_065055734\_6717-model8 | -7.69146 |
|  | XP\_065055734\_6717-model8.1 | -7.00265 |
|  | XP\_065055734\_6717-model8.2 | -6.96056 |
|  | XP\_065055734\_6717-model8.3 | -6.05548 |
|  | XP\_065055734\_6717-model8.4 | -5.34712 |
|  | XP\_065055734\_6717-model8.5 | -4.39901 |
| # cluster 9 - Size: 2 |  |  |
|  | XP\_065055734\_6717-model9 | -6.88261 |
|  | XP\_065055734\_6717-model9.1 | -5.57988 |
| # cluster 10 - Size: 3 |  |  |
|  | XP\_065055734\_6717-model10 | -5.68053 |
|  | XP\_065055734\_6717-model10.1 | -5.14585 |
|  | XP\_065055734\_6717-model10.2 | -4.1748 |
| # cluster 11 - Size: 1 |  |  |
|  | XP\_065055734\_6717-model11 | -5.56546 |
| # cluster 12 - Size: 4 |  |  |
|  | XP\_065055734\_6717-model12 | -4.51686 |
|  | XP\_065055734\_6717-model12.1 | -4.37753 |
|  | XP\_065055734\_6717-model12.2 | -4.06095 |
|  | XP\_065055734\_6717-model12.3 | -4.05156 |
| # cluster 13 - Size: 6 |  |  |
|  | XP\_065055734\_6717-model13 | -4.30042 |
|  | XP\_065055734\_6717-model13.1 | -4.26524 |
|  | XP\_065055734\_6717-model13.2 | -4.2527 |
|  | XP\_065055734\_6717-model13.3 | -4.20721 |
|  | XP\_065055734\_6717-model13.4 | -4.15545 |
|  | XP\_065055734\_6717-model13.5 | -4.03289 |
| # cluster 14 - Size: 2 |  |  |
|  | XP\_065055734\_6717-model14 | -4.25559 |
|  | XP\_065055734\_6717-model14.1 | -4.15067 |
| # cluster 15 - Size: 1 |  |  |
|  | XP\_065055734\_6717-model15 | -4.06016 |
|  |  |  |
